# Detecting Role Errors in the Gene Hierarchy of the NCI Thesaurus

**DOI:** 10.4137/cin.s440

**Published:** 2008-05-27

**Authors:** Hua Min, Barry Cohen, Michael Halper, Marc Oren, Yehoshua Perl

**Affiliations:** 1 Fox Chase Cancer Center, Philadelphia, PA 19111-2497, U.S.A; 2 Computer Science Dept., NJIT, Newark, NJ 07102-1982, U.S.A; 3 Computer Science Dept., Kean University, Union, NJ 07083-0411, U.S.A

**Keywords:** gene terminology, auditing, role error, National Cancer Institute Thesaurus (NCIT), gene hierarchy, taxonomy, abstraction network

## Abstract

Gene terminologies are playing an increasingly important role in the ever-growing field of genomic research. While errors in large, complex terminologies are inevitable, gene terminologies are even more susceptible to them due to the rapid growth of genomic knowledge and the nature of its discovery. It is therefore very important to establish quality-assurance protocols for such genomic-knowledge repositories. Different kinds of terminologies oftentimes require auditing methodologies adapted to their particular structures. In light of this, an auditing methodology tailored to the characteristics of the NCI Thesaurus’s (NCIT’s) Gene hierarchy is presented. The Gene hierarchy is of particular interest to the NCIT’s designers due to the primary role of genomics in current cancer research. This multiphase methodology focuses on detecting role-errors, such as missing roles or roles with incorrect or incomplete target structures, occurring within that hierarchy. The methodology is based on two kinds of abstraction networks, called *taxonomies*, that highlight the role distribution among concepts within the IS-A (subsumption) hierarchy. These abstract views tend to highlight portions of the hierarchy having a higher concentration of errors. The errors found during an application of the methodology are reported. Hypotheses pertaining to the efficacy of our methodology are investigated.

## Introduction

One of the fastest growing areas of biomedical research pertains to knowledge of genes and genomes. The Human Genome Project (HGP) gathered knowledge from human DNA strands and obtained a comprehensive human genome sequence that has strongly impacted many other areas of bioscience and medicine (Collins et al. 1998; Karanjawala and Collins, 1998; Lin, 1998). Advances in our understanding of genomic phenomena are becoming increasingly important for clinical research and medicine as witnessed by the fact that the number of relevant scholarly articles is growing at a staggering rate (Druss and Marcus, 2005). A significant number of databases are now collecting and cataloging genomic data. An annual review of molecular biology databases, for example, lists several hundred databases relevant to the genomic domain (Baxevanis, 2003). The databases that will have the greatest impact are those able to link transparently to other closely related resources.

Thus, gene terminologies are positioned to play a critical role in the area of genomic research. One of the leading terminologies is the Gene Ontology (GO) (The Gene Ontology Consortium 2001) comprising about 19,000 concepts. GO has been integrated into the UMLS (Lomax and McCray, 2004; Sarkar et al. 2003), and has served as the basis for the genomic subcomponents of the National Cancer Institute Thesaurus (NCIT).

While errors in large, complex terminologies are inevitable, gene terminologies are even more highly susceptible to them due to the rapid growth of genomic knowledge and the nature of its discovery. It is therefore very important to establish quality-assurance protocols. In this paper, we present a multiphase, systematic methodology for auditing the genomic component of the NCIT (Covitz et al. 2003; Golbeck et al. 2003; de Coronado et al. 2004; Hartel et al. 2005; Sioutos et al. 2007). The methodology’s focus is on detecting role-errors occurring within the Gene hierarchy. The choice of auditing that hierarchy was made due to the importance of its content in current cancer research. Our methodology is based on two kinds of abstraction networks, called *taxonomies* (originally presented in Min et al. (2006)), that highlight the role distribution among concepts within the IS-A (subsumption) hierarchy. As will be seen, these abstract views tend to highlight areas of the hierarchy having a higher concentration of errors. Various errors, including missing roles and roles with incorrect or incomplete target structures, found during the application of our methodology are reported. Two hypotheses pertaining to the efficacy of the methodology are investigated.

## Background

In this section, we first review the NCIT and its Gene hierarchy. After that, two abstraction networks, called the *area taxonomy* and *p-area taxonomy*, developed in our previous research (Min et al. 2006), are presented.

### Structural characteristics of the NCIT’s gene hierarchy

The National Cancer Institute Thesaurus (NCIT) is a controlled terminology that follows a description logic-based model (Brachman and Levesque, 1984; Brachman and Schmolze, 1985; Nardi and Brachman, 2003) and provides broad coverage of the cancer domain. The basic unit of knowledge in the NCIT is the concept, of which there are 62,352 (June 2007 release). Its concepts include diseases, drugs, chemicals, diagnoses, genes, anatomy, organisms, proteins, and other related subjects (NCICB 2006; NCIT 2006). The IS-A relationship connects a child concept with its parent concept. The NCIT’s concepts are partitioned into 21 separate IS-A hierarchies, including “Biological Process,” “Genes,” “Gene Products,” etc. Roles are lateral relationships between concepts that capture associative, but non-hierarchical, knowledge. All roles of a parent are passed to its child via inheritance.

The Gene hierarchy is of high priority to the designers of the NCIT due to the primary role of genomics in current cancer research.^1^ In this paper, our analysis is based on the 2004 release of the NCIT, whose Gene hierarchy comprises 1,786 concepts. Of these, 1,554 are *leaves* (i.e. have no children). These represent actual genes. The remaining 232 *internal* concepts serve to categorize the genes. The Gene hierarchy is different from other hierarchies of NCIT in that the internal concepts are not themselves gene concepts, but categories of genes. In contrast, an internal concept of the Biological Process hierarchy can be a process with more refined processes as children. For example, the internal concept *Cancer Progression*^2^ describes a process, and has 12 descendants.

There are only 42 concepts with two parents, and all are gene concepts. Examples include *GRB7 Gene*, *MADD Gene*, and *MAGED1 Gene*. Only one of them, *SMARCC2 Gene*, has a child. (This issue of a gene concept with a child is discussed in Section “Discussion”.) The Gene hierarchy has eight levels. An example of a longest path of eight concepts, each one more specific than the previous, is: *Gene*, *Enzyme Gene*, *Hydrolase Gene*, *Phosphatase Family Gene*, *Protein Phosphatase Gene*, *Protein Serine-Threonine Phosphatase Gene*, *Protein Phosphatase 2A Subunit Gene*, and *PPP2R5D Gene*.

The number of children (called the *degree*) for internal (category) nodes varies from 1 to 116, as can be seen in Table 1. For example, *Protein Phosphatase Gene* has 29 descendants, which together make up the protein phosphatase category. Among *Protein Phosphatase Gene*’s five children are three category concepts and two gene concepts. The distribution of the concepts separated into category and gene concepts among the levels of the hierarchy is presented in Table 2. About 83% of the concepts are located on a few middle levels: 73 internal concepts and 500 leaves on Level 2; 68 internal concepts and 409 leaves on Level 3; and 39 internal concepts and 379 leaves on Level 4. We can distinguish between three kinds of category concepts: (1) *Terminal category concepts* where all children are genes; (2) *Generalized category concepts* where all children are also category concepts; and (3) *Mixed category concepts* having both kinds of children. An example of the latter is *Protein Phosphatase Gene*. Table 2 shows that the majority (63%) of categories are terminal, while just a few (5%) are generalized. The remaining 32% are mixed. The 86 generalized and mixed category concepts form the “skeleton” of the Gene hierarchy, providing a compact view of its types of genes.

### The area and p-area taxonomies

In previous work (Min et al. 2006), we have defined the area and p-area taxonomies as abstraction networks for underlying terminology concept networks. Both are derived from the division of a terminology into groups of concepts (*areas* and *p-areas*, respectively) based on their roles and their hierarchical positioning. These taxonomies served as the bases for a structural auditing methodology introduced in Min et al. (2006). It was shown that the taxonomies tend to highlight concept groups with potential errors, and our methodology was successful in uncovering various kinds of errors in the NCIT’s Biological Process hierarchy.

An area is a group of all concepts with the exact same set of roles. Each concept belongs to one and only one area according to its roles. A concept whose parents all belong to areas different from its own is called a *root* of its area. There can be one or more roots in a given area. Those concepts that are not themselves roots will be descendants of the roots.

**Figure 1:**
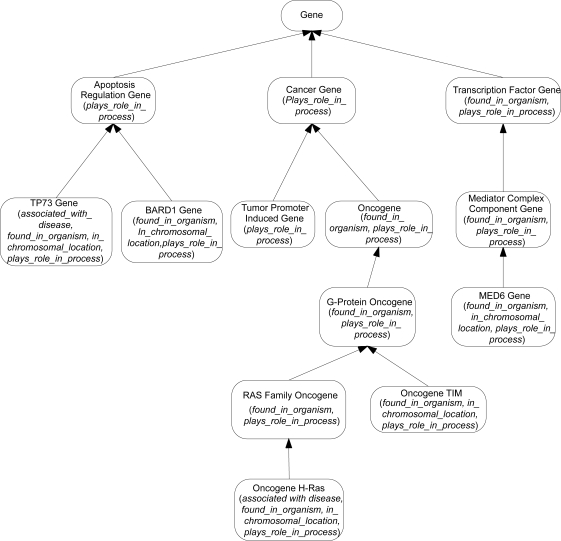
An excerpt of the NCIT Gene hierarchy.

**Figure 2:**
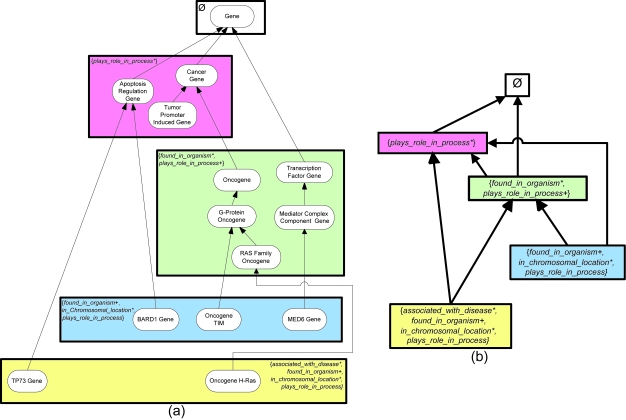
(**a**) Division into areas, and (**b**) area taxonomy.

Let us demonstrate the division of a small excerpt of the NCIT’s Gene hierarchy, shown in Figure 1, into areas. Concepts are drawn as rounded rectangles, with the roles exhibited by each concept listed inside parentheses. The IS-As are represented as arrows directed from the respective child to parent. Figure 2(a) shows the areas derived from this excerpt. An area is denoted as a rectangle that encloses all concepts with the exact same set of roles. An area is named by listing its concepts’ roles in braces. A role is introduced at a concept if it is not defined for any of the concept’s parents. The concepts *Apoptosis Regulation Gene*, *Cancer Gene*, and *Tumor Promoter Induced Gene* with only the role *plays_role_in_process* are grouped together into the area {*plays_role_in_process*}. Concept *Gene*, having no roles, is in the area denoted *φ* (the empty set). The roles *found_in_organism* and *plays_role_in_process* are both introduced at *Transcription Factor Gene*. On the other hand, concept Oncogene inherits *plays_role_in_process* from Cancer Gene and introduces *found_in_organism*. Nonetheless, both Oncogene and Transcription Factor Gene appear in the area {*found_in_organism, plays_role_in_process*}, irrespective of the different ways their roles were obtained.

The area taxonomy (AT) is a hierarchically-structured abstraction network derived from the division of a terminology into areas. Each node in the AT denotes one area. The nodes are connected via *child-of* relationships in a pattern derived from the underlying IS-A links between concepts. Specifically, if a root of an area *X* is a child of any concept in area *Y*, then the AT will have a *child-of* from node *X* to node *Y*. Due to this, the AT is a directed acyclic graph (DAG). Overall, the AT is a concise representation providing a high-level view of the distribution of the terminology’s roles. The area node is an abstraction of a group of concepts with the exact same roles. The *child-of* relationships serve as abstractions of the IS-As.

A root of an area can either (i) strictly introduce all its roles itself (e.g. see concepts *Apoptosis Regulation Gene*, *Cancer Gene*, and *Transcription Factor Gene*), (ii) inherit all its roles from its parents—residing in different areas, or (iii) introduce some roles and inherit others (e.g. *Oncogene*, *Oncogene TIM*, and *Oncogene H-Ras*). One area may have multiple roots that exhibit these different behaviors. Thus, there may be multiple patterns of obtaining roles in an area. We use “*” and “+” to distinguish these various patterns. If a given role is introduced by every root of an area, then that role is followed by a “*” in the area’s name. If a role is inherited by every root of an area, no symbol follows the role’s name. A “+” follows the role name if that role is introduced by some roots and inherited by others. The AT corresponding to Figure 2(a) is shown in Figure 2(b). Note that the area {found_in_organism*, *plays_role_in_process*^+^} is *child-of* both {plays_role_in_process*} and *φ*.

**Figure 3:**
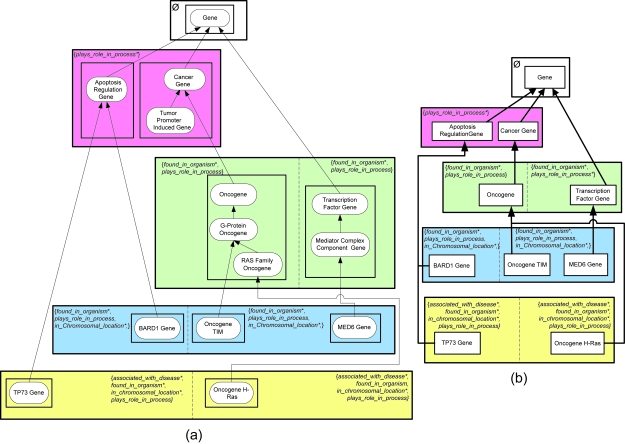
(**a**) Division of areas into p-areas, and (**b**) p-area taxonomy.

If an area has multiple roots, then such an area will be further divided into concept groups based on the roots. A concept collection comprising a root and all its descendants in its area is called a *partial area (p-area)*. For example, concepts *Transcription Factor Gene* and *Oncogene* are two roots of {found_in_organism*, *plays_role_in_process*^+^} because their respective parents *Gene* and *Cancer Gene* do not belong to this area. Area {found_in_ organism*, *plays_role_in_process*^+^} is thus further divided into two p-areas as shown by the solid boxes enclosing *Oncogene* and two of its descendants and *Transcription Factor Gene* and one of its descendants, respectively, in Figure 3(a). Each p-area is named after its unique root. A p-area of one concept is called a *singleton p-area*. Examples of singleton p-areas in Figure 3(a) are *BARD1 Gene*, *Oncogene TIM*, *MED6 Gene*, and *TP73 Gene*.

The division of areas into p-areas leads to an expanded, two-level taxonomy that is called the *p-area taxonomy* (*PAT*). The PAT, similar to the AT, is a DAG, with p-areas represented as nodes and connected to other p-areas via *child-of* relationships. To capture the additional level of division, p-areas are grouped into areas of the AT. The PAT offers a view that provides not only information of the role distribution across the entire terminology but also information of further hierarchical grouping within areas.

The PAT disambiguates the +’s appearing in area names in the AT. Such areas are divided into several collections of p-areas, called *regions*, according to role-introduction patterns, separated from one another in the diagram by a dashed line. Figure 3(b) shows the PAT for Figure 3(a). The area {plays_role_in_process*} has two p-areas, *Apoptosis Regulation Gene* and *Cancer Gene*. For consistency, an area containing just one root is also defined to consist of one p-area, e.g. the p-area *Gene* in the area *φ*. The area {found_in_organism*, *plays_role_in_process*^+^} is divided into two p-areas, in two separate regions, each with a different role-introduction pattern, {found_in_organism*, *plays_role_in_process*} and {found_in_organism*, plays_role_in_process*}.

**Figure 4:**
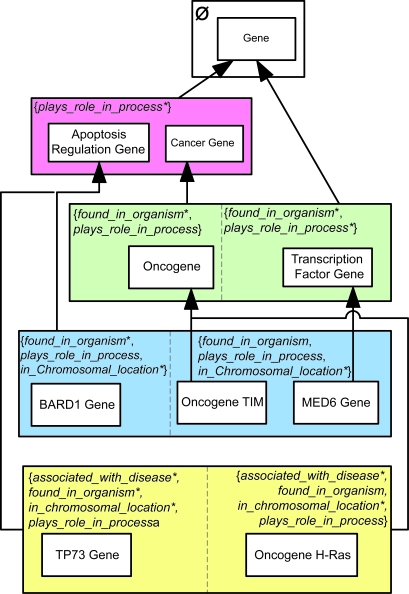
PAT with consolidated *child-of* relationships derived from Figure 3(b).

When there are many small p-areas (as is the case in the PAT of the Gene hierarchy), the *child-of* arrows may cause clutter in the diagram. To avoid this, we group together those p-areas having *child-of* ’s to the same target p-area and consolidate all those *child-of* ’s into one arrow emanating from the group. If the p-areas happen to constitute the entire area (region), then the area (region) box is used as the grouping mechanism. Otherwise, additional boxes are included within the area. See Figure 4 for the consolidated version of Figure 3(b). In particular, note the single arrow emanating from the area {plays_role_in_process*} and directed to the p-area *Gene*.

**Figure 5:**
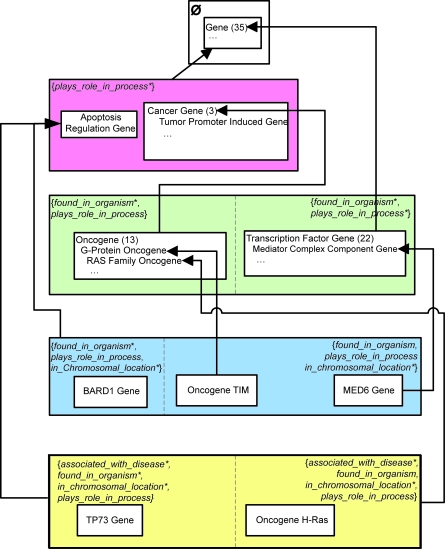
PAT with some concepts displayed in an indented format.

To help in the auditing process, we will at times want to show some or all of the actual concepts residing in a p-area. In such cases, the concepts will be listed in an indented hierarchy format within their p-area box. Examples of this can be seen, for example, in the p-areas *Cancer Gene*, *Oncogene*, and *Transcription Factor Gene* in Figure 5. When included, the number in parentheses alongside a p-area root indicates the total number of concepts in the p-area. Ellipses are used to indicate omitted concepts. In this context, a *child-of* is sometimes drawn directly to a parent concept (not necessarily the root) in the target p-area. An example of this is the *child-of* between *Oncogene TIM* and *G-Protein Oncogene*. Although this does not exactly follow the p-area definition, the more detailed drawing will aid in “group-based auditing,” where, for example, a review of a set of concepts will require review of the concepts’ joint parent in another p-area.

## Methods: Auditing Methodology

Our auditing methodology focuses on the discovery of *role errors* in the Gene hierarchy, not on general modeling errors. Examples of role errors include missing roles, missing targets for existing roles, incorrect targets, and redundant targets. Regarding the latter: if the same role has two target concepts, where one is the parent of the other, then the parent target is redundant because the knowledge conveyed by the role to the child is more refined than that given by the role to the parent. In such a case, the role to the redundant target can be omitted.

The NCIT’s Gene hierarchy defines six different roles: *gene_associated_with disease, gene_found_in_organism, gene_in_chromosomal_location, gene_plays_role_in_process, gene_is_biomarker_type, and gene_is_biomarker_of*.^3^ Nearly all the genes in the NCIT are derived from the DNA sequence data. Therefore, the originating organism and the location of the gene (chromosome and indices of introns) should be known. Also, many genes have known disease-associated alleles. In each such case, the gene should be assigned roles like *gene_associated_with_disease* (in short, “*disease*”), *gene_found_in_organism*, and *gene_in_ chromosomal_location*. Nevertheless, many gene concepts are missing such roles.

Our auditing methodology constitutes manual-review of certain groups of concepts made available automatically to the auditor by the AT and PAT, whose derivation was given in Section “The area and p-area taxonomies”. The tasks of dividing the Gene hierarchy into its areas and p-areas and then deriving the AT and PAT are totally automated. The programs that implement these tasks were written in PERL and comprise less than 1,000 lines of code. The actual graphical forms of the AT and PAT, as seen in the paper, were drawn manually using a software tool. However, their display could very well be automated, too.

We have previously derived a structural auditing methodology based on these abstraction networks, and applied it to the NCIT’s Biological Process hierarchy (Min et al. 2006). However, when conducting the present research, it became clear that the Gene hierarchy is not amenable to the auditing methodology in Min et al. (2006). In particular, that methodology successfully focused on areas with just a few small constituent p-areas. The Gene hierarchy’s structure does not accommodate that kind of approach.

The auditing methodology presented here differs from that in Min et al. (2006) in that it is adapted to the special characteristics of the Gene hierarchy (see Section “Structural characteristics of the NCIT’s gene hierarchy”). For example, with the Biological Process hierarchy (Min et al. 2006), we did not focus on concepts that were leaves. The reason is that one specific process may have another more refined process as its child. For example, *Tumor Angiogenesis* is a child of *Neo-vascularization*. Hence, a process concept may appear in a terminology either as a leaf or as an internal-node concept. But in the Gene hierarchy, no gene concept should be a child of another gene concept. All internal concepts in the Gene hierarchy should represent general categories of genes, e.g. *Tumor Promoter Induced Gene* and *G-Protein Oncogene*. All gene concepts are leaves, appearing as children of the appropriate gene categorization. Since our interest is in auditing the gene concepts for role errors, our attention is focused on the leaves. The research challenge was to design an auditing methodology that fits the Gene hierarchy’s special properties.

**Figure 6:**
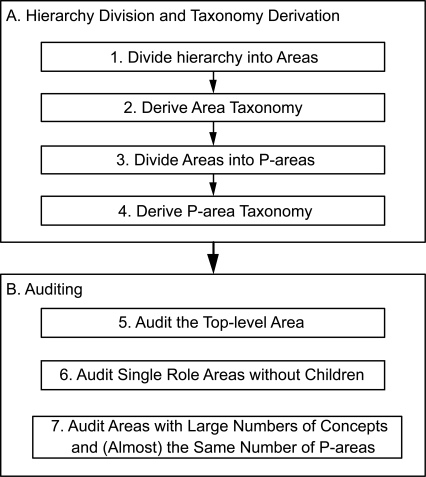
Phases of the auditing methodology.

The major parts of the auditing methodology are summarized in Figure 6. The first major part (A) is the division of the terminology into areas and p-areas and the accompanying derivation of the respective taxonomies. These are shown as Phases 1–4. We would like to emphasize again that these phases are carried out algorithmically.

Part (B) is where the actual auditing takes place. The Phases 5–7 are based on specific concept groups—various areas and p-areas—presented by the AT and PAT. The targeted areas and p-areas are those where the likelihood of finding errors is high. The auditor is directed to focus his or her energy on those portions of the Gene hierarchy. In the following, we describe each of these three phases and the rationale behind their importance.

Let us note that each of the Phases 5–7 constitutes a form of *group-based auditing*, where the auditor is directed to review similar concepts together in the same context rather than as individual, independent concepts. Reviewing a concept in the context of other similar concepts can help expose errors that may not be readily detected otherwise. The AT and PAT are excellent vehicles for this kind of auditing regimen.

### Phase 5: Review of the top-level area *φ*

In previous work (Min et al. 2006), the concepts in the top-level area *φ* have been shown to contain a high percentage of role errors. This is due, in part, to the fact that there is very little semantic similarity among *φ*’s many concepts because there is no true unifying set of roles. There are, in fact, no roles at all. This certainly makes these concepts excellent candidates for missing-role errors.

### Phase 6: Review first-level areas having no children

A first-level area having no child areas and, internally, having only singleton p-areas will only contain concepts that are genes. Moreover, every concept located in a first-level area has just one role. As explained above, knowledge about other roles should be available for genes by their way of discovery. The situation of a gene concept with only one role typically indicates that other roles are missing. On the other hand, if an area of just one role has children, then such an area may contain internal concepts representing categories rather than genes, e.g. *Apoptosis Regulation Gene*, *Chaperone Gene*, and *DNA Repair Gene*, which are less likely to be missing roles.

### Phase 7: Review large areas with large numbers of singleton p-areas

Continuing along the same line of reasoning, one may look for areas with two or more roles that may still be missing other roles. This phase of the methodology concentrates on relatively large areas with all or almost all concepts being genes. Such areas are recognized by having many singleton p-areas. This situation is due to many leaves introducing the same role, which is expected in a gene hierarchy because some roles appear just for genes and not for categories (e.g. chromosomal location). Other roles, such as *gene_plays_role_in_process* (in short, “*process*”) and *disease*, are mainly introduced at the leaf level, although not exclusively. Therefore, the gene concepts tend to appear in singleton p-areas. In a large area with such a configuration, we expect many gene concepts to be missing the same role simultaneously, if they are missing any roles at all.

In fact, to test the efficacy of this phase, we have formulated the following two hypotheses, each expressed in terms of the dependence of the probability of a concept having a role error on its location (i.e. area and p-area) in the PAT.

#### Hypothesis 1

The probability of a given concept having a role error is higher in small p-areas than in large p-areas.

#### Hypothesis 2

The probability of a given concept having a role error is higher in areas with a large number of singleton p-areas than in other areas.

Another two kinds of errors arise in cases where a role exists. One kind is an incorrect target concept for the role. For example, *PPP2R5E Gene* has the chromosomal location 7p12–p11.2 in the NCIT. However, the correct location should be 14q23.1. The other kind of error is the omission of some target(s) for the role. Again, to maximize the number of errors found, while minimizing the effort, this phase focuses on areas with many concepts and many singleton p-areas—a situation indicating many similar gene concepts.

## Results

### AT and PAT for the NCIT gene hierarchy

The Gene hierarchy’s six roles, listed below, form the basis for its division into areas:

gene_associated_with_disease

gene_found_in_organism

gene_in_chromosomal_location

gene_plays_role_in_process

gene_is_biomarker_type

gene_is_biomarker_of

For convenience, these roles will sometimes be designated 0–5, respectively, or abbreviated.

**Figure 7:**
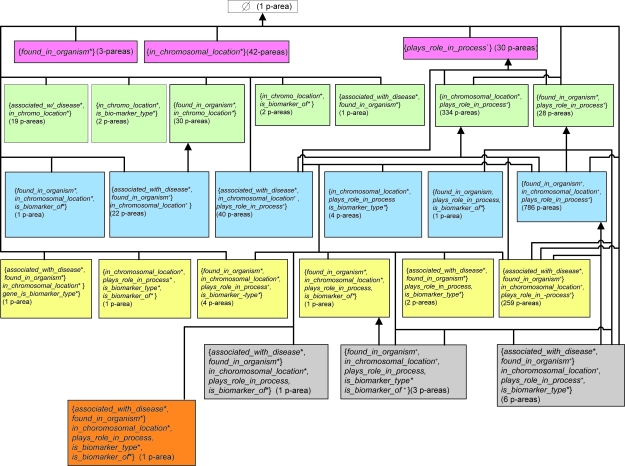
Area taxonomy for the Gene hierarchy.

The AT for the Gene hierarchy is shown in Figure 7, where the prefix “*gene_*” has been omitted from the names of all roles. The 1,786 concepts are divided into 27 areas. As explained in Section “The area and p-area taxonomies”, a “*” following a role name denotes the introduction of that role at the specific area. If the role is inherited from another area, no symbol follows its name. A “+” is used when the role is introduced at some roots and inherited at others. The number in parentheses following the name of an area is its number of p-areas. Areas with the same number of roles are placed on the same level of the AT. Overall, there are seven levels, labeled from (A), the top level, to (G), the lowest level.

**Figure 8:**
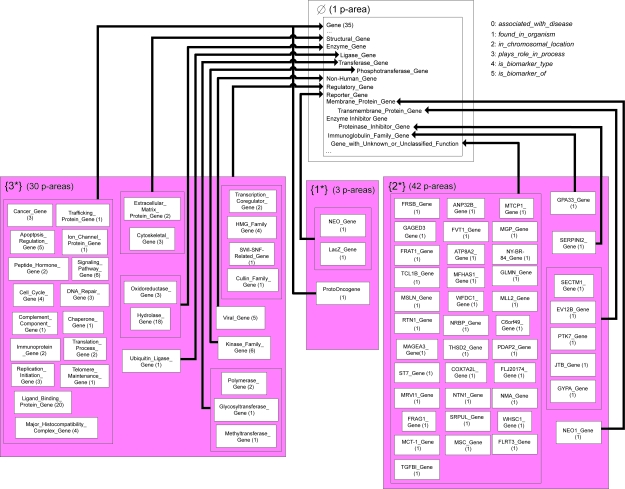
Excerpt of the p-area taxonomy for the Gene hierarchy.

The 27 areas are further divided into a total of 1,583 p-areas. Due to a lack of space, only a portion of the PAT, consisting of the top two levels, (A) and (B), of the AT, is presented in Figure 8. There are four areas from levels (A) and (B) in Figure 8. The number of concepts in a p-area is listed in parentheses following its name. For example, *NEO Gene* and *LacZ Gene* in the area {1*} share a common parent *Reporter Gene* in *φ*. We are using the convention of connecting the p-area directly to its parent instead of using the *child-of* arrow between the p-areas. The “…” in the area *φ* denotes the fact that only those concepts of *φ* that are parents of the roots of p-areas in the four areas are listed.

**Figure 9:**
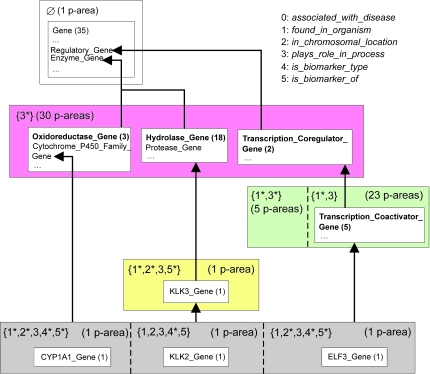
Excerpt of PAT, including areas *φ*, {3*}, {1*, 3^+^}, {1*, 2*, 3, 5*}, and {1^+^, 2^+^, 3, 4*, 5^+^}.

**Figure 10:**
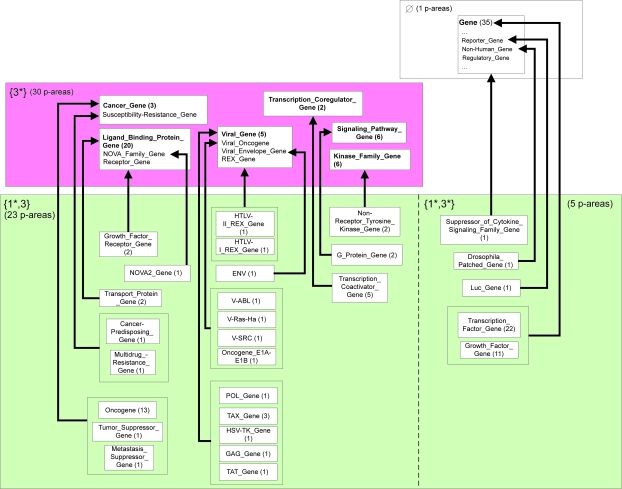
Another excerpt of the PAT.

To demonstrate lower levels of the PAT, we will use the small excerpt shown in Figure 9. The concepts *CYP1A1 Gene*, *KLK2 Gene*, and *ELF3 Gene* have the same roles, 1 through 5, and are grouped into the same area {1^+^, 2^+^, 3, 4*, 5^+^}. The parent *Cytochrome P450 Family Gene* of *CYP1A1 Gene* belongs to {3*}. The parent *KLK3 Gene* of *KLK2 Gene* belongs to {1*, 2*, 3, 5*}. The parent *Transcription Coactivator Gene* of *ELF3 Gene* belongs to {1*, 3^+^}. Since the parents of these three concepts belong to other areas, all three are roots of 1^+^, 2^+^, 3, 4*, 5^+^}. All three roots introduce the role 4, and so * follows 4 in the area’s name. Role 3 is inherited from the parents of the three roots. The root *CYP1A1 Gene* introduces role 1. However, the other two roots, *KLK2 Gene* and *ELF3 Gene*, inherit it. Thus, ‘+’ follows 1 in the area’s name. A similar situation exists for roles 2 and 5. The area is further divided into three p-areas due to its three roots. Those three p-areas have three different introduction patterns for their roles. Thus, they are placed in different regions of the area, separated from each other by dashed lines (Figure 9). All 28 p-areas of {1*, 3^+^} are presented in Figure 10.

The distributions of the areas, p-areas, and concepts among the AT’s levels are summarized in Table 3. For example, level (B) contains three areas, 75 p-areas, and 154 concepts. As we see in Figure 8, the three areas on level (B) introduce the roles 1, 2, and 3, respectively. Level (D) contains the largest number of p-areas among the seven levels. All concepts without any roles are grouped into *φ*, the only area on level (A). We note that the same role can be introduced at different areas on different levels.

A list of all areas along with their numbers of concepts and p-areas is presented in Table 4. The area {1^+^, 2^+^, 3^+^} on level (D) is the largest. It contains 753 p-areas and 778 concepts. Out of the 1,583 p-areas, 1,526 (96%) are singleton p-areas. There are 32 p-areas having two concepts. Table 5 presents the distribution of p-areas and concepts according to p-area size. It shows that the concepts tend to reside in very small p-areas, mainly singleton p-areas. Only 119 concepts reside in p-areas of size greater than ten.

### Role errors discovered

The results of applying auditing Phases (5)–(7) of our methodology to the Gene hierarchy are presented in the following subsections. The reported errors were confirmed based on the National Center for Biotechnology Information (NCBI) Entrez Gene (Entrez Gene 2008; GenBank 2007). A sample of the suggested errors was submitted for review to the editors of the NCIT.

#### Phase (5)

There are 35 concepts in *φ TK Gene* and *CAT Gene* are the only two leaves. *TK Gene* is missing the role 3, while *CAT Gene* is missing roles 2 and 3. The category concept *Enzyme Inhibitor Gene* is missing the *process* role with the target (value) *Enzyme Inhibition. Inhibition* IS-A *Conceptual Entities* in the NCIT, but it should be a process as indicated by the definition and by its semantic type (Natural Phenomenon or Process). Similarly, the three descendants of *Enzyme Inhibitor Gene*, namely, *Proteinase Inhibitor Gene*, *Cysteine Proteinase Inhibitor Gene*, and *Cystatin Superfamily Gene*, are missing this role. Overall, 15 out of *φ*’s total of 35 concepts (43%) are missing roles (see Table 6).

#### Phase (6)

As seen in Figure 7, there are two (B)-level areas without any children: {1*} and {2*}. For area {2*}, all 42 concepts are found to have missing roles! For example, *ANP32B Gene* is missing the role *gene_found_in_organism* with the target *Human*. (This role has been added to the current version of the NCIT.) It should have two roles after the new one is added. Since its parent, *Gene with Unknown or Unclassifi ed Function*, has no roles at all, both should have been introduced at this concept. The concept thus moves from its original area to {1*, 2*}. As another example, *MTCP1 Gene* is missing the roles: *disease* with the target concept *Leukemia; gene_found_in_organism* with the target *Human*; and *gene_plays_role_in_process* with the two targets *Cell Proliferation* and *Regulation of Progression through Cell Cycle*. After adding these three new roles, *MTCP1 Gene* moves to the area {0*, 1^+^, 2^+^, 3^+^}. The required corrections to area {2*}’s other concepts cause them to be moved to a combined five other areas. Thus, {2*} will disappear from a reconstructed AT.

There are only three concepts in {1*}: *NEO gene*, *LacZ gene*, and *Proto-Oncogene. LacZ gene* is missing role 3 (*gene_plays_role_in_process*). It should move to {1*, 3^+^}. On the other hand, *Proto-Oncogene* should not have role 1, and therefore it should belong to *φ*. It is a category concept.^4^ Only the concept *NEO gene* remains in {1*} after the corrections.

Interestingly, the other (B)-level area {3*}, having children (Fig. 7) and not being targeted for auditing in our methodology, is of a different nature from the other two areas. Its concepts are not gene concepts but rather categories of genes (Fig. 8). As a matter of fact, no role errors were observed for any of its concepts (Table 8).

#### Phase (7)

In Phase (7), two criteria determine the areas targeted for auditing. The first is a *large* number of concepts. The second is a *large* number of singleton p-areas. In actually applying this phase, we need now to interpret these criteria quantitatively. We chose the following interpretations: (i) a large area contains more than 30 concepts, and (ii) the ratio of the number of singleton p-areas to the number of concepts should be greater than 0.9. There are nine areas that meet Criterion (i). Three of these nine areas have a ratio less than 0.35. For example, {3*} has 109 concepts but only ten singleton p-areas. There are only six areas that meet both criteria. One such area, {2*}, has already been discussed. The remaining five are {1*, 2*}, {2*, 3^+^}, {0*, 2^+^, 3^+^}, {1^+^, 2^+^, 3^+^}, and {0*, 1^+^, 2^+^, 3^+^} (as shown in Table 4).

The largest area {1^+^, 2^+^, 3^+^} contains 778 concepts and 753 p-areas. Among these, 685 concepts (located in 662 p-areas) have different kinds of errors. For example, the concept *GATA1 Gene* is missing the role *disease* with the target *Dyseryth-ropoietic Anemia*. Seventy-eight concepts have an incorrect chromosomal location. For example, *DTX1 Gene* has chromosomal location 12q24.21. The correct chromosomal location should be 12q24.13. A large number of the concepts (87%) in this area are found to be missing some target values for the *process* role. Although *IL10RB Gene* is connected to *Intercellular Communication* and *Receptor Signaling* via *process*, it is still missing connections to *Blood Coagulation* and *Inflammatory Response*. As this example demonstrates, a single concept may have multiple missing targets with respect to the same role. In fact, in the area {1^+^, 2^+^, 3^+^}, there are 4,616 missing targets for 674 concepts. The multiplicity occurs mainly for the *process* role because the same gene may play a role in many processes.

The areas {0*, 2^+^, 3^+^} and {0*, 1^+^, 2^+^, 3^+^} also have high percentages (98% and 83%, respectively) of missing target values for *process*.

The other two areas have just two roles and have high percentages of missing roles. For example, 305 out of the 339 concepts (90%) in {2*, 3^+^} are missing *gene_found_in_organism*. It also has 27 concepts with the wrong chromosomal location. Furthermore, it has 270 concepts missing targets for *process*. The last area, {1*, 2*}, has 16 out of its 32 concepts (50%) missing roles. Four of its concepts have the wrong chromosomal locations, and 12 concepts are missing targets for *process*.

### Error distributions in p-areas and areas

Role errors were detected for a total of 1,352 concepts. Some of these concepts have more than one kind of role error. There are 390 concepts with missing roles, 1,268 concepts with missing role targets, and 136 concepts with incorrect targets.

Our two hypotheses express expectations for higher probabilities of errors for some specifi c areas and p-areas, targeted for auditing in Phase (7). We now investigate the error distributions across *all* areas and p-areas to check Hypothesis 1 and Hypothesis 2 and establish the effectiveness of our approach.

The distribution of erroneous concepts by p-area size is presented in Table 7. Singleton p-areas, for example, accounted for 1,286 erroneous concepts, which amounts to 84% of their constituent concepts (see row 1 in Table 7). P-areas with two concepts exhibited a total of 36 erroneous concepts (56%). (For another kind of error, see Section “Discussion”.) The error percentage goes down as the size of the p-areas gets above two, though no specific trend is seen. However, when all errors are counted together for those p-areas, the error percentage (15%) is significantly smaller than that for singleton p-areas and two-concept p-areas (83%, gleaned from the first two rows of Table 7). This result confirms Hypothesis 1.

The distribution of errors among areas is presented in Table 8. In addition to the number of erroneous concepts (column 4) and their respective percentages (column 5), the table includes the numbers of erroneous concepts and percentages with respect to each kind of role error. The last column shows the total number of missing targets for existing roles with respect to a given area. For the Gene hierarchy as a whole, there are 8,570 missing targets for 1,268 concepts—on average, more than six missing targets per concept. For the five areas selected by the second criterion discussed in Section “Phase (7)” of the Results, the percentages of erroneous concepts are as follows: 50% for {1*, 2*}; 90% for {2*, 3^+^}; 98% for {0*, 2^+^, 3^+^}; 88% for {1^+^, 2^+^, 3^+^}; and 83% for {0*, 1^+^, 2^+^, 3^+^}. Hypothesis 2 is confirmed by the fact that in total 87% of the concepts in these five areas have role errors.

The other two areas, {1*} and {2*}, with just one role and without any children also show high error percentages: 67% for {1*}, and 100% for {2*}. When combining these two areas, 44 out of 45 concepts (98%) have errors. All the concepts in these areas are gene concepts. This result confirms the viability of auditing Phase (6) regarding such concepts. The top-level area *φ* has a 43% error rate, confirming the viability of Phase (5). Altogether, in the areas targeted by Phases (5)–(7) of our auditing methodology, 86% of the concepts have role errors. For comparison, in all other areas combined, only 14% of the concepts have such errors.

## Discussion

### Interpretation

The AT and PAT were derived from divisions of the Gene hierarchy based on structural and positional similarity of concepts. The AT has 27 areas, which is two orders of magnitude smaller than the underlying Gene hierarchy network (1,786 concepts). However, the PAT comprises 1,583 p-areas, almost as many as the number of concepts. This is mainly due to roles introduced at gene concepts, which are the leaves of the hierarchy. Thus, only the AT, not the PAT, serves as a compact abstraction network. But both taxonomies have been successfully employed in automatically presenting the auditor with portions of the Gene hierarchy that are ripe for investigation concerning role errors.

As we have seen, many concepts in the Gene hierarchy are missing roles. For example, the 42 concepts in the area {2*}, each having just role 2, are all missing at least one role. With so many roles missing from the Gene hierarchy, the partition into areas is not accurate enough to support general-purpose, taxonomy-based auditing. Thus, we took the approach of first dealing strictly with role errors. Such role errors, e.g. a concept with a missing role, will tend to be highlighted and consequently more easily detected. In this paper, we reported on our efforts in detecting such errors. Only after correcting the roles could auditing for other errors proceed, as the revised partition into areas would then be more reliable.

For both hypotheses, we used an empirical approach for the interpretation of the “small” and “large” values. At the same time, we explored changes to these values and their impact. Hypothesis 1 asserts that the probability of erroneous concepts is higher for small p-areas than for large p-areas. When interpreting small p-areas as those having one or two concepts, we get a collective error percentage of 83% in such p-areas. The error percentage decreases to 15% for larger p-areas, which supports Hypothesis 1. Thus, it is fruitful to concentrate the auditing efforts on small p-areas of one or two concepts. Phase (7) focuses on areas having many such p-areas. Note that increasing the “small” threshold to three or even four has negligible effect. P-areas of size three have a low error percentage (15%) as compared to generally larger p-areas, and those of size four have no errors at all (see Table 7).

As a side remark, we found another kind of error that was prevalent in two-concept p-areas: incorrect IS-A relationships. In those cases, the p-area consisted of two gene concepts, one an IS-A of the other. This arrangement violated the foundational rule of the Gene hierarchy that all gene concepts be leaves and all internal nodes be category concepts. Thus, a gene concept should only be a child of a category concept. All errors of this kind except one were corrected independently by the NCIT editorial team in the 2006 release of the NCIT. The one such error still appearing is: *IICER1 Gene* IS-A *RNase 3 Gene*.

Hypothesis 2 asserts that the probability of erroneous concepts is higher in areas with large numbers of concepts and large numbers of singleton p-areas than in other areas. When we interpret large areas as those containing at least 30 concepts, the percentage of erroneous concepts is 86%. If, on the one hand, we lower the “large” threshold to 15—which captures two additional areas, {0*, 2*} and {0*, 1^+^, 2^+^}, with a total of 13 erroneous concepts—the error percentage is just slightly reduced to 85%. If, on the other hand, we raise the threshold to 100 concepts—and thereby lose areas {1*, 2*} and {0*, 2^+^, 3^+^} and their 55 erroneous concepts—the percentage is hardly changed at 87%. The conclusion is that any reasonable threshold value for interpreting “large number of concepts” will suffice.

Overall, there were three kinds of role errors found in the NCIT’s Gene hierarchy: missing roles (i.e. complete absences of roles that should exist), incorrect chromosomal location roles, and missing target concepts for the multi-valued process roles. In the last case, one or more processes were missing, while a set of processes was already associated with a specific gene. As an evaluation, we compared our findings (suggested corrections) with the current (07.12e) version of the NCIT, for which the Gene hierarchy had undergone a major re-design in 2006.^5^ We discovered the following:

- Concerning the missing roles, there were two genes with missing chromosomal locations: *cat gene*, which in the 07.12e release has the same location *11p13* as we suggested, and the *tat gene*, which still has no such role in the latest release. There were a total of 346 genes missing the organism role, 61 missing the process role, and 17 missing the disease role. Out of these, 55, 38, and 10, respectively, now have the associated organisms, processes, and diseases that we suggested in our report. We note that only 390 concepts that were missing roles are listed in Table 8. This is due to the fact that a single gene may have been missing several roles.
- 117 out of the 136 genes that previously had incorrect chromosomal locations now have corrected locations that are the same as our suggestions.
- Concerning the most frequent error that we reported—process roles with missing targets—NCIT 07.12e did not change much. In a sample of 100 genes missing a total of 815 processes, we found only eight of our corrections regarding such missing target processes. It was probably the case in the re-design of the NCIT in 2006 that most of the attention was paid to the missing role errors where the mistakes were obvious. Also, the focus was probably on the incorrect chromosomal locations that could be observed when comparing the NCIT with other sources such as Entrez Gene (Entrez Gene 2008) and OMIM (OMIM 2008).^5^ The least attention was likely given to the missing processes for genes already having some associated processes.

### Movement of concepts to different areas

Out of the three kinds of role errors, only “missing role” affects the area of a concept. A concept that has been corrected by giving it additional roles effectively moves from its original area to the area where the concepts exhibit the revised set of roles. The correction of a “missing target” or “incorrect target” does not have any impact on a concept’s area since the role existed already and additional or different targets have no bearing on areas.

There were 390 concepts with missing roles. The pattern of their movement to new areas is shown in Table 9. For example, 14 of *φ*’s concepts move to {3*}. Another moves to {2*, 3^+^}. An extreme case is area {2*}, where all 42 of its concepts change areas. These new areas are: {0*, 2*}, {1*, 2*}, {1^+^, 2^+^, 3^+^}, {0*, 1^+^, 2^+^}, and {0*, 1^+^, 2^+^, 3^+^} (Table 9). The area {2*}, in fact, would not be part of a reconstructed AT.

### Limitations

The role of auditing is to report potential errors and recommend corrections. However, only the terminology’s curators, who know the design policy used in modeling the terminology and have a broad perspective on its whole content, have the authority to actually make the corrections. What may look like an error to an auditor may look correct to a curator. Thus, all errors reported in this paper should be taken as *potential* errors unless acknowledged by the NCIT’s curators. With regard to the gene concepts’ roles, there is the extra complication concerning the reliability of publications reporting on the involvement of a gene in, say, a disease or biological process. There certainly may be cases where the report of such a finding is not considered reliable enough by the NCIT’s curators to warrant inclusion in the terminology. Furthermore, it seems that the editors of the NCIT employ stricter reliability criteria than those used for the Entrez Gene database (Entrez Gene 2008) of NCBI.^6^ In Entrez Gene, the knowledge is annotated with a variety of evidence codes (EMBL 2007). Such evidence needs to be considered by the NCIT’s curators when judging alleged errors.

We note that auditing methodologies may vary from one terminology to another depending on the underlying structure. While the framework of the taxonomy-based auditing introduced in Min et al. (2006) is promising for terminologies with a design similar to the NCIT hierarchies, fine-tuning may be required for specific hierarchies. In fact, the methodology presented herein has been tailored to the Gene hierarchy. Phase (5) and Hypothesis 1 were also used in Min et al. (2006) in the context of the Biological Process hierarchy, but Phases (6) and (7) and Hypothesis 2 have not appeared previously. Let us stress the difference between Hypothesis 2 and its counterpart used in Min et al. (2006). Here, we concentrated on small p-areas in large areas, while in Min et al. (2006), we focused on small p-areas in small areas. Due to such differences, it is important to examine the structural properties of a target terminology, as was done for the Gene hierarchy in Section “Structural characteristics of the NCIT’s gene hierarchy”.

### Generalizability and future work

As we noted, the structure of the Gene hierarchy is different from that of other NCIT hierarchies in that all gene concepts are leaves. Another NCIT hierarchy following such a design is Gene Product,^7^ which mainly models proteins. Hence, the adaptations of the framework of Min et al. (2006) for the Gene hierarchy may be applicable to that hierarchy, too. The fact that these two hierarchies are of high priority to the NCI due to their importance in cancer research^6^ warrants such adaptation efforts.

We note that our taxonomy-based auditing approach has also proven fruitful for auditing SNOMED CT (IHTSDO 2007) as demonstrated in Wang et al. (2007). Due to this, it will accommodate terminologies such as Kaiser Permanente’s Convergent Medical Terminology (CMT) (Dolin et al. 2004) derived from SNOMED CT. Moreover, SNOMED’s design has anticipated the need for extensions and subsets in order to construct terminological artifacts that are fine-tuned for particular hospitals and other such organizations—and groups of organizations. The purpose of SNOMED’s “reference set specification” (College of American Pathologists 2006) is to allow for the extraction of SNOMED components that are tailored to specific organizational preferences and use-cases. Given this and the recent purchase of SNOMED CT by the IHTSDO, more derived terminologies of the SNOMED ilk that are amenable to our taxonomy-based auditing techniques can be expected in the future.

There has been a trend toward standardization in the field of terminologies and ontologies. See, for example, the activities of the ISO (International Organization for Standardization 2000). There is also OBO (The Open Biomedical Ontologies 2008), which now comprises over 60 biomedical ontologies (Smith et al. 2007), including NCIT itself. It is a challenge to explore ways to adapt the taxonomy-based auditing framework to support auditing many more of these ontologies.

## Conclusion

A multiphase auditing methodology has been applied to the Gene hierarchy of the NCIT in an effort to uncover role errors, including omitted roles and those with incorrect or incomplete target structures. The Gene hierarchy was divided into collections of concepts called areas and p-areas, from which two abstraction networks, the area taxonomy and p-area taxonomy, were derived. These taxonomies helped prioritize auditing efforts by revealing groups of concepts with a high likelihood of the role errors we were after. The auditing conducted according to our methodology found that about 75% of the concepts in the Gene hierarchy exhibit role errors. The error distributions have been reported. The collective error percentage in small p-areas (having one or two concepts) is much higher (83%) than for larger p-areas (15%), confirming a proposed hypothesis. The error percentage for large areas having many singleton p-areas is high (above 50%), confirming another hypothesis of ours. After correcting the role errors, the newly corrected hierarchy is more reliable in capturing groups of concepts with similar sets of roles. This corrected hierarchy is then ready to be audited again using other suitable methodologies in the search for other kinds of errors, e.g. incorrect or missing IS-As.

## Figures and Tables

**Table 1:** Degree distribution for internal (category) concepts of the Gene hierarchy.

Degree	# Internal concepts	Degree	# Internal concepts	Degree	# Internal concepts
1	56	12	2	31	1
2	40	13	1	35	2
3	19	14	3	36	3
4	17	15	2	38	1
5	14	16	1	47	1
6	12	17	2	50	1
7	10	20	1	51	1
8	9	21	2	67	1
9	9	22	2	74	1
10	6	23	1	89	1
11	8	24	1	116	1

**Table 2:** Characteristics of the Gene hierarchy’s levels.

Level	# Generalized category concepts	# Mixed category concepts	# Terminal category concepts	Total # category concepts	# Gene concepts	Total # concepts
0	0	1	0	1	0	1
1	2	20	8	30	6	36
2	4	24	45	73	500	573
3	4	18	46	68	409	477
4	1	7	31	39	379	418
5	1	4	10	15	126	141
6	0	0	6	6	102	108
7	0	0	0	0	32	32
**Total:**	**12**	**74**	**146**	**232**	**1,554**	**1,786**

**Table 3:** Distribution of areas, p-areas, and concepts by level.

Level	# Areas	# P-areas	# Concepts
(A)	1	1	35
(B)	3	75	154
(C)	7	413	476
(D)	6	821	846
(E)	6	262	264
(F)	3	10	10
(G)	1	1	1
**Total:**	**27**	**1,583**	**1,786**

**Table 4:** Areas with their numbers of concepts and p-areas.

Area	# Concepts	# P-areas
*φ*	35	1
{1*}	3	3
{3*}	109	30
{2*}	42	42
{0*, 1*}	1	1
{0*, 2*}	19	19
{1*, 2*}	32	30
{1*, 3^+^}	81	28
{2*, 3^+^}	339	331
{2*, 4*}	2	2
{2*, 5*}	2	2
{0*, 1^+^, 2^+^}	22	22
{0*, 2^+^, 3^+^}	40	40
{1^+^, 2^+^, 3^+^}	778	753
{1*, 2*, 5*}	1	1
{1, 3, 5*}	1	1
{2*, 3, 4*}	4	4
{0*, 1^+^, 2^+^, 3^+^}	255	253
{0*, 1*, 2*, 4*}	1	1
{0*, 2*, 3, 4*}	2	2
{1*, 2*, 3, 4*}	4	4
{1*, 2*, 3, 5*}	1	1
{2*, 3*, 4*, 5*}	1	1
{0*, 1^+^, 2^+^, 3^+^, 4*}	6	6
{0*, 1*, 2*, 3, 5*}	1	1
{1^+^, 2^+^, 3, 4*, 5^+^}	3	3
{0*, 1*, 2*, 3, 4*, 5*}	1	1
**Total:**	**1,786**	**1,583**

**Table 5:** P-area size distribution.

P-Area size	# P-areas	Total # concepts
1	1,526	1,526
2	32	64
3	9	27
4	3	12
5	4	20
6	3	18
11	1	11
13	1	13
18	1	18
20	1	20
22	1	22
35	1	35
**Total:**	**1,583**	**1,786**

**Table 6:** Concepts missing roles in *φ.*

Concept name	Missing role	Role target value
TK Gene	3	Phosphorylation
CAT Gene	2	11p13
	3	Detoxification, Acetylation
Enzyme Inhibitor Gene	3	Enzyme Inhibition
Proteinase Inhibitor Gene	3	Enzyme Inhibition
Cysteine Proteinase Inhibitor Gene	3	Enzyme Inhibition
Cystatin Superfamily Gene	3	Enzyme Inhibition
Enzyme Gene	3	Biochemical Reaction
Ligase Gene	3	Biochemical Reaction
Transferase Gene	3	Biochemical Reaction
Phosphotransferase Gene	3	Biochemical Reaction
Regulatory Gene	3	Biochemical Process
hGH Gene	3	Biochemical Process
Nucleosome Assembly Protein Gene	3	Biochemical Process
Immunoglobulin Gene	3	Host Defense Mechanism
CEA Family Gene	3	Host Defense Mechanism

**Table 7:** Erroneous concept distributions by size of p-area.

P-area size	# P-areas	Total # concepts	# Erroneous concepts	% Errors
1	1,526	1,526	1,286	84%
2	32	64	36	56%
3	9	27	4	15%
4	3	12	0	0%
5	4	20	5	25%
6	3	18	6	33%
≥7	6	119	15	13%
**Total:**	**1,583**	**1,786**	**1,352**	**76%**

**Table 8:** Error distributions among areas and p-areas.

Area	# Concepts	# P-areas	Concepts w/errors		P-areas w/errors		Concepts w/missing roles		Concepts w/wrong chromosomal location		Concepts w/missing targets		Total # missing targets
			#	%	#	%	#	%	#	%	#	%	
*φ*	35	1	15	43	1	100	15	43	-	-	1	3	7
{1*}	3	3	2	67	1	33	1	33	1	33	-	-	-
{2*}	42	42	42	100	42	100	42	100	3	7	27	64	103
{3*}	109	30	-	-	-	-	-	-	-	-	-	-	-
{0*, 1*}	1	1	-	-	-	-	-	-	-	-	-	-	-
{0*, 2*}	19	19	3	16	3	16	3	16	-	-	2	11	6
{1*, 2*}	32	30	16	50	16	53	12	38	4	13	12	38	31
{1*, 3^+^}	81	28	1	1	1	4	1	1	-	-	1	1	8
{2*, 3^+^}	339	331	305	90	300	91	305	90	27	8	270	80	1,904
{2*, 4*}	2	2	1	50	1	50	1	50	-	-	1	50	20
{2*, 5*}	2	2	1	50	1	50	1	50	-	-	1	50	3
{0*, 1^+^, 2^+^}	22	22	10	45	10	45	8	36	2	9	8	36	25
{0*, 2^+^, 3^+^}	40	40	39	98	39	98	-	-	2	5	39	98	237
{1^+^, 2^+^, 3^+^}	778	753	685	88	662	88	1	-	78	10	674	87	4,616
{1*, 2*, 5*}	1	1	-	-	-	-	-	-	-	-	-	-	-
{1, 3, 5*}	1	1	-	-	-	-	-	-	-	-	-	-	-
{2*, 3, 4*}	4	4	3	75	3	75	-	-	-	-	3	75	17
{0*, 1^+^, 2^+^, 3^+^}	255	253	212	83	212	84	-	-	18	7	212	83	1,487
{0*, 1*, 2*, 4*}	1	1	-	-	-	-	-	-	-	-	-	-	-
{0*, 2*, 3, 4*}	2	2	2	100	2	100	-	-	-	-	2	100	15
{1*, 2*, 3, 4*}	4	4	4	100	4	100	-	-	-	-	4	100	45
{1*, 2*, 3, 5*}	1	1	1	100	1	100	-	-	-	-	1	100	3
{2*, 3*, 4*, 5*}	1	1	1	100	1	100	-	-	-	-	1	100	1
{0*, 1^+^, 2^+^, 3^+^, 4*}	6	6	5	83	5	83	-	-	1	17	5	83	22
{0*, 1*, 2*, 3, 5*}	1	1	1	100	1	100	-	-	-	-	1	100	3
{1^+^, 2^+^, 3, 4*, 5^+^}	3	3	2	67	2	67	-	-	-	-	2	67	16
{0*, 1*, 2*, 3, 4*, 5*}	1	1	1	100	1	100	-	-	-	-	1	100	1
**Total:**	**1,786**	**1,583**	**1,352**	**76**	**1,309**	**83**	**390**	**22**	**136**	**8**	**1,268**	**71**	**8,570**

**Table 9:** Movement—after corrections—of concepts previously found to have missing roles.

Concept’s original area	Concept’s new area	# Concepts moved
*φ*	{3*}	14
*φ*	{2*, 3^+^}	1
{1*}	{1*, 3^+^}	1
{2*}	{0*, 2*}	1
{2*}	{1*, 2*}	12
{2*}	{1^+^, 2^+^, 3^+^}	14
{2*}	{0*, 1^+^, 2^+^}	9
{2*}	{0*, 1^+^, 2^+^, 3^+^}	6
{0*, 2*}	{0*, 2^+^, 3^+^}	3
{1*, 2*}	{1^+^, 2^+^, 3^+^}	12
{1*, 3^+^}	{1^+^, 2^+^, 3^+^}	1
{2*, 3^+^}	{1^+^, 2^+^, 3^+^}	305
{2*, 4*}	{2*, 3, 4*}	1
{2*, 5*}	{2*, 3*, 5*}	1
{0*, 1^+^, 2^+^}	{0*, 1^+^, 2^+^, 3^+^}	8
{1^+^, 2^+^, 3^+^}	{0*, 1^+^, 2^+^, 3^+^}	1
**Total:**		**390**

## Footnotes

1 F.W. Hartel, personal communication.

2 A capitalized italic font is used for concepts.

3 Role names will be italicized and start with a lowercase letter.

4 Dr. Nicole Thomas, personal communication.

5 G. Fragoso, personal communication.

6 F.W. Hartel, personal communication.

7 G. Fragoso, personal communication.
